# A GEANT4 Monte Carlo simulation study for the computation of output factors and out-of-field dosimetry in breast IOERT

**DOI:** 10.1038/s41598-025-31407-1

**Published:** 2025-12-07

**Authors:** Sara Savatović, Licia Toscano, Mara Severgnini, Francesco Longo

**Affiliations:** 1 Research Group Biomedical Imaging Physics, Department of Physics, TUM School of Natural Sciences & Munich Institute of Biomedical Engineering, Technical University of Munich, 85748 Garching, Germany; 2 Department of Physics and Astronomy “Galileo Galilei”, University of Padua, 35131 Padua, Italy; 3 CRO Aviano, National Cancer Institute, IRCCS, 33081 Aviano, Italy; 4 Department of Medical Physics, Azienda Sanitaria Universitaria Giuliano-Isontina (ASUGI), 34129 Trieste, Italy; 5 Department of Physics, University of Trieste, 34127 Trieste, Italy; 6 National Institute for Nuclear Physics (INFN), Sezione di Trieste, 34127 Trieste, Italy

**Keywords:** IOERT, Mobetron1000, GEANT4, MC simulation, 3D dosimetry, Output factor, Computational biology and bioinformatics, Medical research, Physics

## Abstract

The goal of breast Intraoperative Electron Radiation Therapy (IOERT) is to deliver a uniform single fraction dose of 10–25 Gy to a surgically exposed volume of tissue at risk (tumor bed) while minimizing exposure to surrounding healthy tissue, with the patient under anesthesia. Currently, all the necessary information for the geometric and physical treatment arrangement is collected during the commissioning phase, when a preliminary dosimetric characterization of the accelerator is performed for clinical use in accordance with international protocols. During this process, output factors (OFs)—correction factors that account for dose differences relative to the reference condition—are measured. Therefore, since treatment plans rely exclusively on the OF and experimental measurements can be affected by large errors (on the order of 30%), a tuned Monte Carlo model provides a valuable tool for treatment validation. It is particularly valuable for evaluating quantities that are difficult to measure directly, such as dose variations resulting from shielding disk misalignment (to protect underlying healthy tissue) or out-of-field exposure to organs at risk in complex clinical scenarios (e.g., patients with implantable electronic devices or during pregnancy).

## Introduction

Intraoperative Radiation Therapy (IORT) is a specialized form of radiation therapy used in the treatment of cancer. It involves delivering a single high dose of radiation to a specific target area within the body during surgery at the time of the tumor removal. IORT is often used to treat specific types of cancer, especially when the tumor is adjacent to vital structures or organs and traditional External Beam Radiation Therapy (EBRT) may not be as effective or may affect nearby healthy tissues^1^. Different IORT modalities are available in clinical practice, including electrons, low-kV X-rays, and high-dose-rate brachytherapy^2^. When electrons are employed, the technique is referred to as Intraoperative Electron Radiation Therapy (IOERT). The Mobetron1000 linear accelerator (LINAC) employed in this study delivers electrons at three operative energies, namely 6 MeV, 9 MeV and 12 MeV.

In the conventional approach, post-surgery EBRT is administered for most tumor types to eradicate any residual microscopic disease and to reduce the risk of local recurrence^2,3^. Accurately pinpointing the location of the tumor bed can be challenging, and in some cases, larger treatment margins may be necessary. This can increase the risk of dose deposit to healthy tissues^3^. However, even with advanced EBRT techniques designed to precisely conform to the tumor site, limitations exist in the dose that can be safely delivered due to the presence of nearby organs at risk or critical structures (e.g., pancreatic cancer^4^). In such cases, IOERT can be considered as an option due to its steeper dose gradient when delivering large doses. High-energy electrons deposit their energy over a limited range, delivering the dose locally, whereas X-rays penetrate more deeply into tissue^3^. Moreover, the shape and size of the radiation field can be controlled through the appropriate selection of the applicator and the use of shielding disks, thereby sparing the surrounding healthy tissue.

IOERT is often delivered as a single-dose treatment, administered immediately after tumor resection, minimizing potential dose delivery issues caused by intrafraction motion in EBRT^5^. However, it is not suitable for all cancer types or patients, and is typically reserved for specific cases where it can provide an effective treatment outcome. The decision to use IOERT is made on a case-by-case basis, with the patient’s medical team considering various factors, including the type and stage of cancer, the location of the tumor, and the patient’s overall health^6,7^.

In particular, breast IOERT is commonly prescribed as a single exclusive intraoperative dose of 21 Gy, typically delivered either to the 90% isodose covering the tumor bed or to the point of maximum dose deposition ($z_{\textrm{max}}$), which was used as the prescription point in this study. In some cases, IOERT may be delivered as a boost of 10 Gy, followed by EBRT^8^. The dose prescription is determined by the radiotherapy oncologist, and guidelines recommend covering the target within a 10% variation from the prescribed dose^9^. Patient selection remains a critical factor in the success of breast IOERT, as demonstrated by different clinical trials conducted since the initial ELIOT trial in 2013^10^. More recent studies emphasize that the selection of eligible candidates should be more stringent than the criteria defined in the suitable category of the ASTRO guidelines^11^, to ensure favorable clinical outcomes and reduce the risk of local recurrence^12^.

In clinical practice, dose calculations are typically performed in real time during surgery, taking into account the depth electrons must traverse to achieve adequate coverage of the tumor bed. This consideration guides the choice of applicator and electron beam energy, as well as the potential use of a tissue-equivalent bolus on the tumor bed surface. Differences exist among the shapes of the isodose curves for different IOERT machines (e.g., Mobetron, NOVAC, LIAC^9^) and in non reference conditions in IOERT^13,14^. These differences arise as a result of different collimation systems that the accelerators employ. In fact, the collimation system and the air column above the patient cause an angular dispersion of the beam as well as an energy spread. Applicators with different diameters and angled edges (beveled applicators) produce distinct dose distributions.

To account for these differences, output factors (OFs) are used. The OFs are defined as the ratio of the dose $D_{\mathrm {non-reference}}(z_{\textrm{max}})$ measured at $z_{\textrm{max}}$ for a given applicator and energy, relative to the dose $D_{\textrm{reference}}(z_{\textrm{max}})$ measured with the 10 cm flat applicator at the same energy. Where $z_{\textrm{max}}$ represents the depth of maximum dose along the central axis for the given energy. The reference electron penetration characteristics correspond to those obtained with a 10 cm flat applicator for each energy^13^. Experimental OFs may vary due to measurement uncertainties^15^. Therefore, a validated MC simulation provides a valuable tool for their verification. Although previous MC studies, using EGSrc, have addressed dosimetric aspects of IORT implementation for the Mobetron1000 from a clinical perspective, simulations using Penelope2008/GEANT4 have so far been limited to radiation protection studies^16^. In this study, we provide simulated OFs from a tuned GEANT4 MC model in of the Mobetron1000, supporting clinical decision-making and further dosimetric studies.

Breast tissue is mainly uniform, and thus using OFs is a reasonable choice, since a heterogeneous tissue would affect the final dose distribution^14,17^. In such cases, MC simulations can address this limitation, and several MC-based approaches have been developed as in-house solutions^18–21^. To our knowledge, no commercial MC Treatment Planning System (TPS) for IOERT is currently available. An ideal TPS for IOERT should incorporate real-time imaging techniques, such as CT scans or ultrasound, as recently reported in^21,22^, to enable precise localization of the tumor or tumor bed and surrounding structures. Alternatively, postoperative dosimetry can be performed using MC simulations^20^. However, it is limited by anatomical changes that occur between imaging and irradiation, which may affect the accuracy of the dose assessment. Dose deviations from the intended prescription have been reported in literature, reaching up to 34% in breast irradiation and over 300% in bone compared to water^15,17^, highlighting the need for reliable dose calculation, especially in the proximity of realistic anatomical regions characterized by substantial tissue inhomogeneity (i.e., soft tissue, air, bone).

In breast IOERT, a shielding disk is placed beneath the tumor bed and above the rib cage during irradiation to protect underlying organs^23^. Previous MC studies have investigated the design of shielding disks and applicators and their influence on dose distribution, these analyses were limited to the 12 MeV electron beam^24,25^. The positioning and design of the shielding disk can affect the dose received by organs at risk, especially the heart and lungs, if the disk is not optimally aligned with the applicator. A local, immediately next to the shielding disk, dosimetric characterization was performed in previous studies for disk misplacement^24^. However, to date, no simulated dose values delivered to these organs have been reported in the literature. In this work, we provide simulated organ doses, offering insights into the potential risks associated with suboptimal disk placement.

## Materials and methods

GEANT4 is a toolkit for the simulation of the transport of particles through matter^26^. In this work, it was used to study dose distributions in a water phantom, as well as for the estimation of out-of-field dose deposited to a computational anthropomorphic phantom^27^. A scientific computing network service, INFN-farm.ts, was used to reduce the elapsed simulation time by parallelizing tasks across multiple nodes. The reference physics list used in the simulation was QGSP_BIC_HP_PEN, which includes the particle transport physics recommended for radiation protection purposes and energies below 200 MeV (QGSP_BIC), a higher precision neutron model for energies $\le 20$ MeV (HP) and a lower energy electromagnetic physics builder model (PEN). The main transport parameters were set according to the GEANT4 (v10.1.p02) default configuration for this physics list: production cuts of 1 mm for electrons, positrons and photons, an electron tracking energy threshold of 100 eV, and a step limitation defined by a range factor of 0.2 and a final range of 1 mm. For neutrons, the HP transport models tracked down particles down to thermal energies ($1\times 10^{-5}$ eV).

The MC model was tuned in successive steps to reproduce the experimental beam characteristics under reference conditions (10 cm, 0$^{\circ }$-bevel applicator). First, the accelerator geometry was implemented according to the manufacturer’s specifications, under a non-disclosure agreement. Subsequently, the source was tuned for each nominal energy, iterating over the source size, energy distribution, and divergence. The performance of each iteration was quantitatively evaluated using a gamma index analysis, comparing the simulated profiles, at the reference depth, with measured ones. The gamma index provides a numerical quality index that serves as a measure of disagreement in the regions that fail the acceptance criteria and indicates the calculation quality in regions that pass^28^. In this study, the measured curves were considered as the reference (expected) distribution, while the simulated curves were treated as the evaluated (tested) distribution, following the approach commonly used in clinical TPS commissioning for conventional LINACs^29^. The analysis was done for a global tolerance of $3\,\textrm{mm}/3\%$, following AIFM (Associazione Ialiana Fisica Medica), ESTRO (European Society for Radiotherapy and Oncology) guidelines^30,31^ and previous work^32^. No threshold was applied for the low doses. And finally, a phase-space plane, capable of recording particle information, was introduced in the MC simulation to record the energy, position, and angular distribution of primary electrons after the scattering system for each nominal energy to calculate OFs for non-reference applicators. Out-of-field dose, and dose comparison in case of shielding disk misalignment in the MIRD phantom was done by adding the full LINAC’s head into the human phantom GEANT4 example. The MIRD phantom is one of the earliest and simplest computational phantoms available in GEANT4. Nevertheless, it still offers a reasonable geometrical representation and realistic physical properties of human organs.

### Geometry

**Fig. 1:**
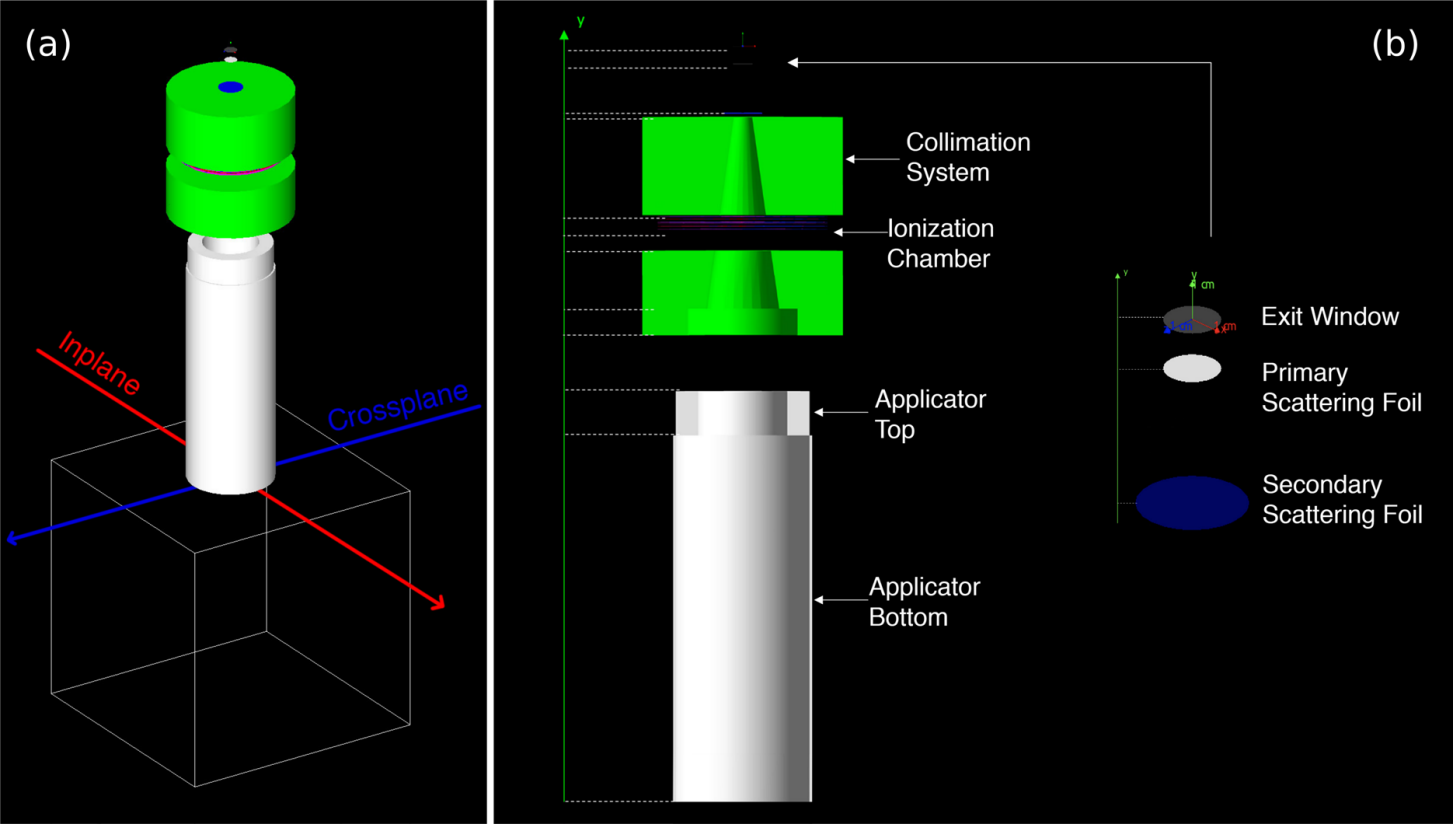
LINAC’s representation in GEANT4 with the reference, non-beveled applicator, 10 cm **in diameter.** The voxelized phantom outline is shown for an easier visualization. **(a)** Accelerator above the water phantom. **(b)** Accelerator main components and applicator parts.

The LINAC’s geometry was built following the manufacturer’s specification sheet. A schematic representation of the LINAC is shown in Fig. 1. The Mobetron1000 consists of an initial beam broadening system, comprising the exit window and the dual-foil scattering filter system, a collimation system, an ionization chamber and the applicator, which has a thicker structure with alignment mirrors on the top and a cylinder at the bottom. The size and bevel of the applicator can be easily changed by the user in the simulation through an external configuration file.

A voxelized water phantom, resembling the one used during the commissioning and periodical quality checks, was positioned underneath the LINAC’s geometry and is shown in Fig. 1a. Its dimensions are $30\times 30\times 30\,\hbox {cm}^{3}$, containing $3\times 10^{6}$ sensitive volume elements with voxel size $1\times 1\times 1$ $1\times 1\times 1\,\hbox {mm}^{3}$. Each voxel recorded information on the type of incident particle and the deposited energy in such volume.

### Source

Most linear accelerators produce a narrow electron beam, typically a few mm in diameter, which is essentially Gaussian in profile. The beam is then widened by introducing a scattering foil system. The Mobetron1000 uses a dual-foil system (see Fig. 1b). Even though the simulation was built according to the manufacturers guidelines, there were still some ambiguity in the description of the composition for few of the LINAC element’s alloys. The Percentage Depth Dose (PDD) curves and dose profiles changed substantially by modifying the Secondary Scattering Foil’s (SSF) alloy properties.

**Fig. 2:**
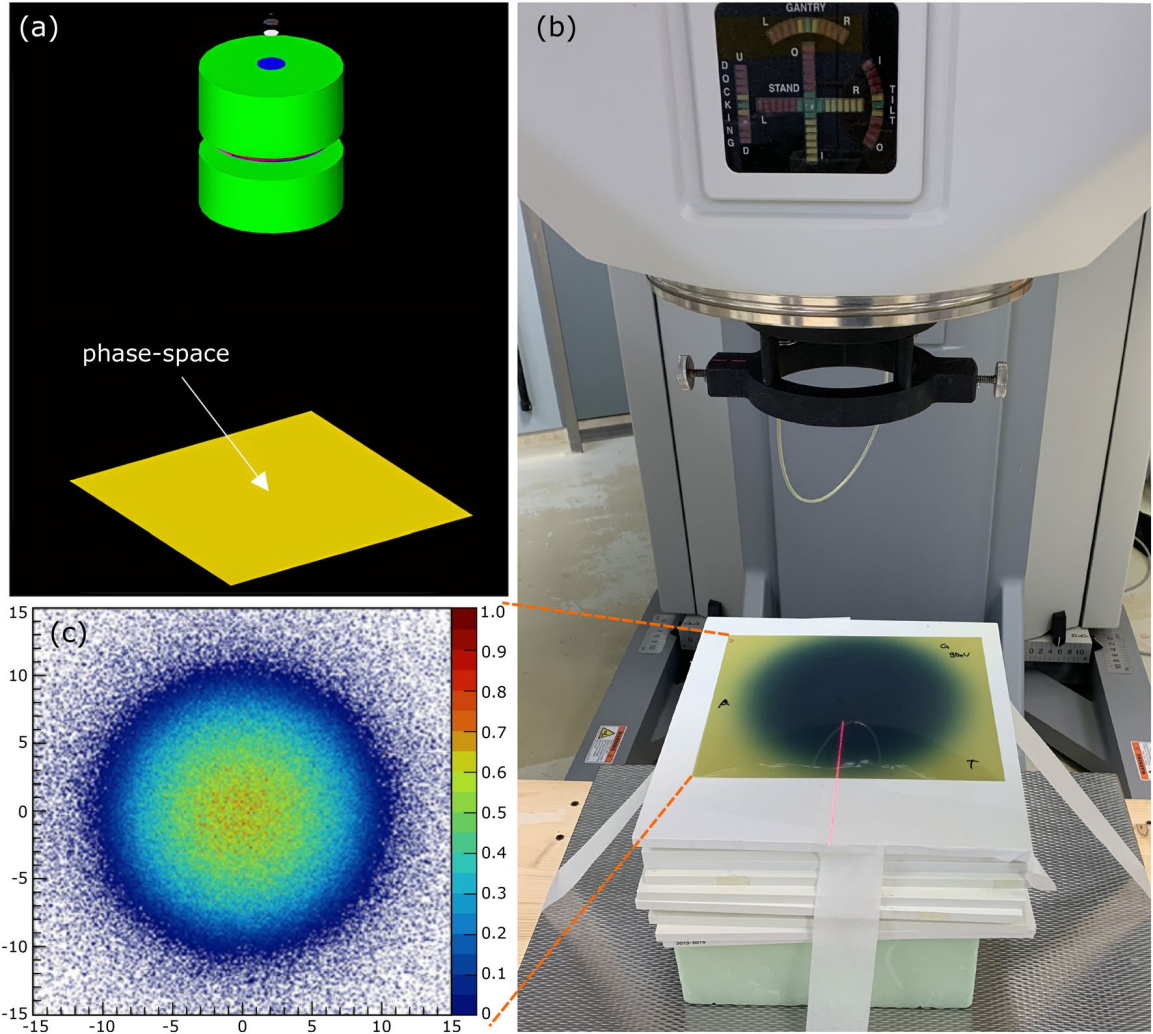
Phase-space—sensitive geometry element in the simulation used to record particle properties. **(a)** shows the simulated geometry. The circular source Gaussian intensity distribution, with a spot FWHM of 1.6 mm for $2\times 10^{6}$ generated 9 MeV primary electrons, is shown in **(c)**. **(b)** Irradiated GAFchromic film, without any applicator. A GAFchromic film was placed at $z_{\textrm{max}}=18\,\textrm{mm}$ between the layers of the PTW solid water slab phantom at the reference depth. The top layers of the slab phantom are removed to display the film in **(b)**.

The source parameters were tuned to reproduce the measured beam characteristics under reference conditions. The simulation geometry was defined according to the manufacturer’s specifications (see Fig. 1), and the source model parameters, initially set to the manufacturer’s values, were iteratively adjusted to best match the experimental data. In particular, an irradiation profile was acquired without any applicator to provide a reference starting point for the tuning process. A GAFchromic EBT-3 dosimetry film (Ashland, Wilmington, DE, USA) was used to acquire such profile for the 9 MeV beam at a reference depth in a solid water phantom RW3 Slab Phantom (PTW Freiburg GmbH, Freiburg, Germany), as shown in Fig. 2. This dataset was essential for optimizing the source parameters, including the SSF alloy density, spot size, energy distribution ($\overline{E}$,$\sigma _E$), and angular divergence.

### Simulation tuning

A sensitive plane, or phase-space, was implemented in the MC simulation to record particle state variables—such as particle type, position, direction, and energy—in order to derive the beam profile at a specific depth. Figure 2 shows the simulated beam profile (c) at the same depth in the water phantom and at the same distance from the source as the measured profile (b) for a 9 MeV beam.

The tuning process started with the 9 MeV electron beam and the reference measurement without the applicator. As the initial profiles exhibited over-pronounced shoulders and a central dip compared to the measurements, the density of the SSF was slightly adjusted to soften the central fluence distribution and achieve a closer match before optimizing the source and energy parameters, which alone were not sufficient to reproduce the measured profile. Once these profiles matched, the simulation was fine-tuned in successive steps using the voxelized water phantom and reference curves measured during the commissioning for all the energies:

1. *Initial setup* The beam energy and geometry were defined following the Mobetron1000 technical documentation.
2. *Mean energy and energy spread determination* The mean energy ($\overline{E}$) and standard deviation ($\sigma _E$) of the Gaussian energy distribution were iteratively adjusted in steps of 0.05 MeV and 0.01 MeV, respectively, around the values reported in the manufacturer’s specifications to match the reference measurements. Agreement between experimental and simulated data was evaluated using gamma index analysis with a 3 mm/3% acceptance criterion, requiring at least 90% of the points to meet the condition.
3. *Source size and divergence tuning* Once the energy parameters were fixed, the source spot size and angular divergence were refined. The source spot diameter was varied between 0.5 to 8 mm, and the angular divergence between 0$^{\circ }$ to 5$^{\circ }$. The best agreement was achieved for a circular source with a Gaussian intensity distribution, with equal widths in both transverse directions (FWHM = 1.6 mm) and no initial divergence.
4. *Final parameter selection* The parameters were then simulated in the reference commissioning measurement condition with the voxelized phantom. First for the 9 MeV beam and later for the others. Repeating step 2 and 3 iteratively until more than 95% of the points verified the gamma criterion.

The tuning in step 4 was done by performing a gamma index analysis between the simulated and measured PDDs, inplane and crossplane dose profiles at the reference $z_{\textrm{max}}$ for every nominal beam energy (13 mm, 18 mm, and 22 mm for the 6 MeV, 9 MeV, and 12 MeV, respectively) for the reference applicator. To evaluate the simulated dose distributions, a scanning volume comparable to the dosimetry Diode E (PTW Freiburg GmbH, Germany) detector, used in commissioning measurements, was employed. A sensitive volume of $1\times 3\times 6\,\hbox {mm}^{3} = 18\,\hbox {mm}^{3}$ was used for the profile evaluation, and a scoring volume of $1 \times 6 \times 6\,\hbox {mm}^{3} = 36\,\hbox {mm}^{3}$, similar to that of the Advanced Markus (PTW Freiburg GmbH, Germany) parallel-plate ionization chamber, was adopted for the assessment of the PDDs. The experimental data collected during commissioning were sampled with a step size of 1 mm for both PDDs and profiles, except in the central flat region of the profiles, where the step size was 4 mm (interpolated to 2 mm for the gamma evaluation), and in the PDDs beyond the buildup, where it was 2 mm.

After reproducing the reference curves in the simulation, the phase-space plane was moved beneath the collimation system (above the applicator, see Supplementary Fig. S1) and phase-space datasets were recorded for each nominal energy. These datasets were then used to simulate non-reference applicators and to compute the corresponding OFs.

## Results

The optimal mean energies and corresponding $\sigma _E$ values are listed in Table 1. These values are consistent with previous MC studies of the Mobetron1000 system using EGSrc^32,33^. Furthermore, the PDDs and dose profiles derived from this model showed the closest agreement with measurements even under more complex configurations (e.g., small or beveled applicators).

**Table 1:** Nominal operative and simulated source energy.

Nominal energy [MeV]	Simulated mean energy $\overline{E}$ [MeV]	$\sigma _E$ [MeV]
6	7.2	0.7
9	9.8	0.9
12	12.35	1.2

Similar values can be found in literature^32,33^.

**Fig. 3:**
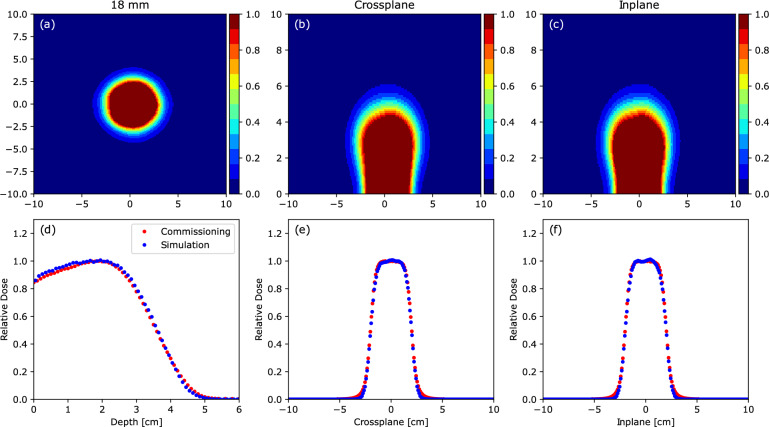
Simulation output for a 9 MeV electron beam. Simulated water phantom dose distribution for the small field with the 4 cm 0$^{\circ }$-bevel applicator (blue) and experimentally measured data (red). The isodose distributions are normalized to the central axis dose at $z_{\textrm{max}}=18\,\textrm{mm}$ [center in **(a)**]. Isodose distributions in the inplane and crossplane planes are shown in **(b,c)**, along with their dose profiles at $z_{\textrm{max}}$ in **(e,f)**, respectively. The PDD is shown in **(d)**.

**Fig. 4:**
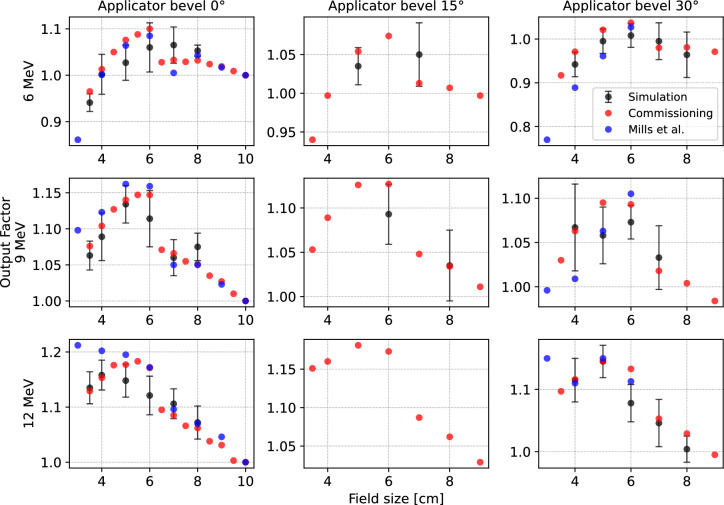
Output factor comparison. The graphs show simulated OF values (black), experimental measurements acquired during commissioning (red), and literature data from Mills et al. ^13^ (blue). Results are presented for the three operative energies: 6 MeV, 9 MeV, and 12 MeV, from top to bottom. The horizontal axis in the plots represents the applicator diameter or field size, while columns from left to right correspond to beveled applicators at 0$^{\circ }$, 15$^{\circ }$, and 30$^{\circ }$, respectively.

Figure 3 compares the simulated and measured data for a 9 MeV electron beam with the 4 cm 0$^{\circ }$-bevel applicator. In the graphs, the red dots represent the experimental measurements, while the blue dots correspond to the simulation results. The gamma index analysis was performed for all beam configurations (combinations of energy and applicator) for which an OF was estimated (see Fig. 4). Among these, the lower operative energies yielded better results, with more than 95% of the points passing the gamma criterion. In comparison, over 91% of the points satisfied the gamma criterion for the 12 MeV beam. Some fluctuations remain in the profiles, particularly for the reference applicator and 9 MeV beam (see Supplementary Fig. S2) and are less prominent in the other energies. These may be attributed to residual statistical uncertainties or systematic effects.

Neutron models were included to account for potential contamination from high-Z elements in the LINAC head, as in conventional radiotherapy^34^. However, their contribution to the total deposited energy was negligible, consistent with the low neutron yield expected for 12 MeV electrons in the Mobetron1000 head and with previous studies and measurements on IOERT accelerators^15,16^. In the 12 MeV beam phase-space file, only about a hundred of neutrons were recorded out of more than $10^{9}$ entries. Therefore, neutron dose evaluation was not further pursued, as improving the statistics would have required excessive computation time beyond the scope of this study.

### OFs comparison

In IOERT, correction factors have to be applied to account for differences in dose distribution. The prescribed dose has to account for air gaps, beam obliquity, and possible tissue heterogeneities. Here, the validated simulation allowed us to cross-check the measured field OFs and compare them to literature^13^ as shown in Fig. 4. The simulated OFs were assessed by observing the deposited energy in the central axis voxel at the reference depth for every nominal energy for a non reference applicator diameter and bevel. The deposited energy is proportional to the absorbed dose, therefore the OF can be calculated as

1 $$\begin{aligned} \textrm{OF}=\frac{D_{\mathrm {non-reference}}(z_{\textrm{max}})}{D_{\textrm{reference}}(z_{\textrm{max}})} \, , \end{aligned}$$

where:

- $D_{\textrm{reference}}(z_{\textrm{max}})$ is the maximum dose in the central beam axis, corresponding to the depth $z_{\textrm{max}}$ in reference conditions (see Table 7.II^35^, applicator diameter 10 cm);
- $D_{\mathrm {non-reference}}(z_{\textrm{max}})$ is the maximum dose delivered in the central beam axis, corresponding to the depth $z_{\textrm{max}}$ in non-reference conditions.

Figure 4 shows a comparison between the OF obtained from the simulation (black) and experimental measurements (red). Moreover, the graphs show values found previously in literature^13^ (blue). Relative differences between the different OFs can be found as Supplementary Table S1 online. Error bars for the simulation were estimated based on the number of deposited energy from particle entries in each voxel and by propagating the uncertainties in Eq. (1). Fewer OFs were simulated for the 15$^{\circ }$-bevel applicators, as the 0$^{\circ }$ applicators are more commonly used. In addition to the standard field OFs, a separate correction factor (OF$_\mathrm {{air-gap}}$)^13^ should be applied when an air gap exists between the tissue and the applicator. Preliminary results for the corrective air-gap OF$_\mathrm {{air-gap}}$ for 1 cm ($0.970\pm 0.036$) and 0.5 cm ($0.989\pm 0.049$) of air for a 6 MeV beam using the reference applicator showed good agreement with the experimental measurements (0.972 and 0.958, respectively).

### 3D dose distribution: anthropomorphic phantom & disk alignment

**Fig. 5:**
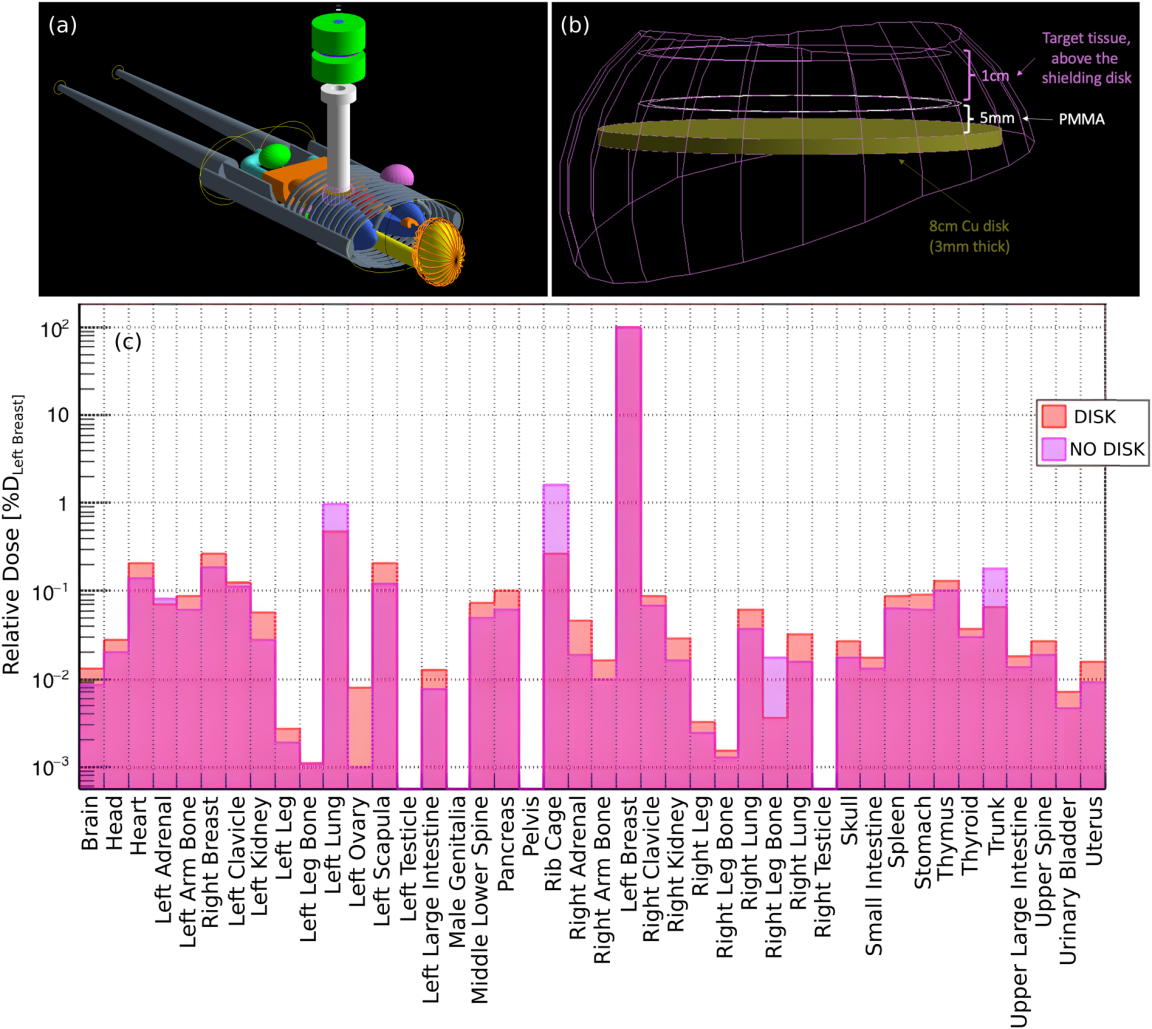
Representation of the geometry in the simulation for a left breast IOERT treatment. **(a)** Adaptation of the human phantom example’s breast geometry to resemble a realistic target structure. **(b)** Left breast irradiation whit the copper shielding disk inserted inside the computational phantom’s geometry. **(c)** Dose to different organs in the computational phantom for a 9 MeV beam. All the accessible volumes are shown; however, male-specific organs, which were not simulated and therefore received zero dose, can be disregarded.

Once the simulation was validated and the source tuned to known specifications, the LINAC’s structure was introduced in the GEANT4 human phantom advanced example, shown in Fig. 5a. A female MIRD phantom^27^ was used to estimate the out-of-field organ dose. The goal was to assess the dose to healthy organs, such as the lungs or heart, in the event of shielding disk misalignment during breast IOERT. The simulation geometry was designed to resemble a typical left breast treatment, with the Mobetron1000 positioned above the left breast. The target geometry was adjusted to replicate a realistic scenario, as shown in Fig. 5b. We simulated a 6 cm $0^{\circ }$-bevel applicator and an 8 cm-diameter shielding disk positioned beneath the target, which is 1 cm thick. An identical simulation, without the shielding disk, was created to study the case of complete misalignment and assess the maximum dose to the underlying organs. The simulation outputs a root file, containing information on the energy deposited in each sensitive volume identified by an organ ID number. The deposited energy $E_{\textrm{organ}}$ in the organ can be then converted to mean dose as follows:

2 $$\begin{aligned} D_{\textrm{organ}}=\frac{E_{\textrm{organ}}}{m_{\textrm{organ}}} \, , \end{aligned}$$

where $m_{\textrm{organ}}$ is the organ mass, calculated using the organ’s volume and density.

The relative dose in $D_{\textrm{LeftBreast}}\%$ delivered to various body parts of the phantom with correct shielding disk alignment is presented in Table 2. Figure 5c shows the percentage of “Left Breast” dose $D_{\textrm{LeftBreast}}$ to different organs on a semi-logarithmic plot. The main differences in the dose delivered to organs with and without the shielding disk are found in the rib cage (from $0.27 D_{\textrm{LeftBreast}}\%$ to $1.60 D_{\textrm{LeftBreast}}\%$), trunk (from $0.07 D_{\textrm{LeftBreast}}\%$ to $0.178 D_{\textrm{LeftBreast}}\%$), and left lung (from $0.47 D_{\textrm{LeftBreast}}\%$ to $0.99 D_{\textrm{LeftBreast}}\%$), whereas other variations of relative dose to the left breast dose result negligible or of the order of statistical fluctuations (0.2% for the left breast dose). It follows that, due to the electron beam short range, the dose, in the case of complete shield misalignment, has a major increase in the rib cage and trunk.

**Table 2:** Relative dose $D_{\textrm{LeftBreast}}\%$ (in percentage) to the phantom’s body parts with the correct shielding disk alignment.

Organ	Dose	Organ	Dose
Brain	$0.013\pm 5.9\%$	Left Breast	$100.0\pm 0.2\%$
Head	$0.028\pm 2.3\%$	Right Clavicle	$0.088\pm 19\%$
Heart	$0.210\pm 3.0\%$	Right Kidney	$0.029\pm 13\%$
Left Adrenal	$0.070\pm 35\%$	Right Leg	$0.003\pm 4.5\%$
Left Arm Bone	$0.087\pm 2.5\%$	Right Leg Bone	$0.002\pm 15\%$
Right Breast	$0.273\pm 3.6\%$	Right Lung	$0.061\pm 4.7\%$
Left Clavicle	$0.125\pm 16\%$	Right Ovary	$0.004\pm 213\%$
Left Kidney	$0.056\pm 9.2\%$	Right Scapula	$0.032\pm 12\%$
Left Leg	$0.003\pm 5.0\%$	Skull	$0.027\pm 4.5\%$
Left Leg Bone	$0.001\pm 17\%$	Small Intestine	$0.018\pm 6.2\%$
Left Lung	$0.472\pm 1.7\%$	Spleen	$0.090\pm 6.6\%$
Left Ovary	$0.008\pm 144\%$	Stomach	$0.092\pm 4.3\%$
Left Scapula	$0.209\pm 4.6\%$	Thymus	$0.132\pm 14\%$
Lower Large Intestine	$0.013\pm 13\%$	Thyroid	$0.037\pm 38\%$
Middle Lower Spine	$0.074\pm 2.9\%$	Trunk	$0.067\pm 0.5\%$
Pancreas	$0.102\pm 11\%$	Upper Large Intestine	$0.018\pm 9.3\%$
Rib Cage	$0.270\pm 1.6\%$	Upper Spine	$0.027\pm 12\%$
Right Adrenal	$0.046\pm 43\%$	Urinary Bladder	$0.007\pm 46\%$
Right Arm Bone	$0.016\pm 5.8\%$	Uterus	$0.016\pm 25\%$

Uncertainties represent relative percentage errors for each value, emphasizing the reliability of the estimated relative dose for each organ, which depend on the organ volume and the deposited energy.

The lungs are among the most radiosensitive organs and are vital for sustaining life. In this study, data show that a single IOERT treatment—prescribing 21 Gy at the $z_{\textrm{max}}$—results in a mean dose to the left lung of about ($100\pm 1.7$)mGy, as estimated from Table 2, when the shielding disk is correctly positioned and aligned with the electron beam. In the absence of the shielding disk, the dose to the left lung approximately doubles. It is important to note, however, that this analysis estimates the dose to the entire organ. To obtain a more detailed picture of the spatial dose distribution within an organ, a dose-volume histogram (DVH) would be required. Although a DVH was not evaluated in this study, it could be derived by dividing the organ of interest into smaller sub-volumes and analyzing the deposited energy within each. In future work, adopting more detailed voxelized phantoms could improve anatomical accuracy and enable a more comprehensive evaluation of individual organ dose distributions.

The peripheral dose to different organs can be assessed from Table 2, by assuming that $D_{\textrm{LeftBreast}}\%=21$ Gy is imparted to the left breast with correct shielding disk alignment. The dose to the heart result to be about $(440.0\pm 13.2)$mGy while $(3.36\pm 0.84)$mGy are delivered to the uterus in a single boost IOERT treatment. This result is of major importance for the evaluation of the feasibility for pregnant women with breast cancer to undergo conservative breast surgery followed by a single boost IOERT.

## Discussion and conclusion

IOERT is employed in the treatment of several tumor types, including breast cancer^6,7^, pancreatic cancer^36^, colorectal cancer^37^, and sarcomas^38^, among others^2,9^. Here, we focused on breast cancer treatment^11^, which is routinely performed at the Cattinara hospital, Trieste, Italy^39^.

In this study, we built the simulation to focus on two key points: first, the implementation and validation of the MC simulation of the Mobetron1000 for the computation of field OFs, and second, the estimation of out-of-field dose to various organs within a computational phantom.

Using the MIRD phantom, we were able to estimate doses to major organs and evaluate the potential out-of-field exposure, while the simplified anatomy limits the spatial resolution and prevents capturing intra-organ dose variations. The use of voxelized or patient-specific computational phantoms would enable a more realistic and spatially detailed dose evaluation, accounting for organ heterogeneity and local dose gradients more accurately than the simplified MIRD model. Moreover, such phantoms would allow the reconstruction of organ dose–volume histograms (DVHs). Although more advanced computational models are available in GEANT4, they generally require substantially higher memory capacity and computational resources^40^. For this reason, the MIRD phantom was adopted as a first approximation for organ dose estimation, while the use of more anatomically detailed phantoms can be explored in future work.

The major drawback of the MC method is the computing time required to obtain an acceptable statistical uncertainty in the simulated quantities. In our case, with $30\times 10^{6}$ generated primary electrons, the beam profile relative uncertainty in the central region was about 2%, which is comparable to the experimental uncertainty 2.1% reported in Ref.^15^.

Our findings demonstrate that the dose to the fetus is minimal in the case of a pregnant patient undergoing breast IOERT. This is due to the treatment’s localized distribution, suggesting that the procedure can be considered safe for both the patient and the fetus. Although breast cancer is rarely diagnosed in pregnancy (about 1 per 1000). In such cases, mastectomy is the usual treatment, conservative surgery is not an option because the postoperative radiotherapy must be delayed until after parturition. Other studies^41^ performed in-vivo dosimetry with thermoluminescence radiation detectors (TLD)s to estimate the dose to the uterus during IOERT, and they have found similar results (in the range 0.6–3.2 mGy). Several estimates of the peripheral dose to the uterus from photon fields in breast cancer EBRT have been published in literature, ranging from 50 mGy to more than 250 mGy to the shielded or unshielded fetus^42^. Hence EBRT is typically not recommended during gestation, since it could cause detrimental health effects to the fetus. The International Commission on Radiological Protection (ICRP) considers a fetal dose of less than 1 mGy to be insignificant, and that doses of a few mGy are acceptable as they are associated with no measurable increased risk of fetal damage^42^, hence breast IOERT could be proposed as an alternative to mastectomy for pregnant woman with breast cancer, as Galimberti et al.^41^ suggest. The report^42^ emphasizes that mammalian embryos and fetuses exhibit a high degree of radiosensitivity. The type and extent of biological effects triggered by radiation exposure depend on both the radiation dose and the specific fetal developmental stage at which exposure occurs. Hence, the need for a precise assessment of the dose can help in evaluating the feasibility of the treatment. The fetal radiation dose, for breast IOERT treatment, estimated in this study aligns with the guidelines provided in the NCRP Report No. 174^43^ (i.e., biological effects $< \mathrm {0.10}\,Gy$ can be considered negligible).

Breast IOERT is typically administered in a single session during lumpectomy surgery, greatly simplifying local therapy. This reduces the overall treatment duration compared to EBRT, which was traditionally delivered over many fractions spanning several weeks and is now often administered in a hypofractionated schedule of five or more fractions^44,45^. The shorter treatment schedule can be especially advantageous for patients who may be considered frail or sensitive^46^. However, we stress that the treatment plan should be determined through collaboration between the patient, the surgical team, and the radiation oncology team to ensure the most appropriate and effective approach for the individual patient. Since these treatment schemes are often used in controlled clinical studies, ensuring dosimetric accuracy is crucial for the reliability of the clinical information expected to emerge from such direct/experimental treatment regimens. An important technological advancement would be the integration of in-room real-time imaging, supported by treatment planning systems capable of incorporating 3D data, real-time dose calculation, adaptive planning, and in-vivo treatment verification.

In conclusion, we successfully simulated the Mobetron1000 linear accelerator using GEANT4 and validated the model across all operative energies, with over 90% of points meeting the gamma index criterion of 3%/3 mm. Using this validated setup, we computed output factors for different applicator diameters and bevel angles across all energies, providing essential data for accurate treatment planning. We also estimated out-of-field dose to various organs using an anthropomorphic phantom and assessed dose levels in the event of complete shielding disk misalignment during breast IOERT. Notably, the dose to the uterus was found to be extremely low, suggesting that, under controlled conditions, breast IOERT could be considered even for pregnant patients.

## Supplementary Information

Supplementary Information.

## Data Availability

Data presented in the study are available from the corresponding author, S.S., on reasonable request.
