# Supplementary material for: A GEANT4 Monte Carlo simulation study for the computation of output factors and out-of-field dosimetry in breast IOERT
Source: Sci Rep. 2025 Dec 7;15:43270. doi: 10.1038/s41598-025-31407-1 (PMC12686418; doi:10.1038/s41598-025-31407-1)
Supplement: Supplementary file 1 — Supplementary Information. [file 41598_2025_31407_MOESM1_ESM.pdf]

Supplementary Information

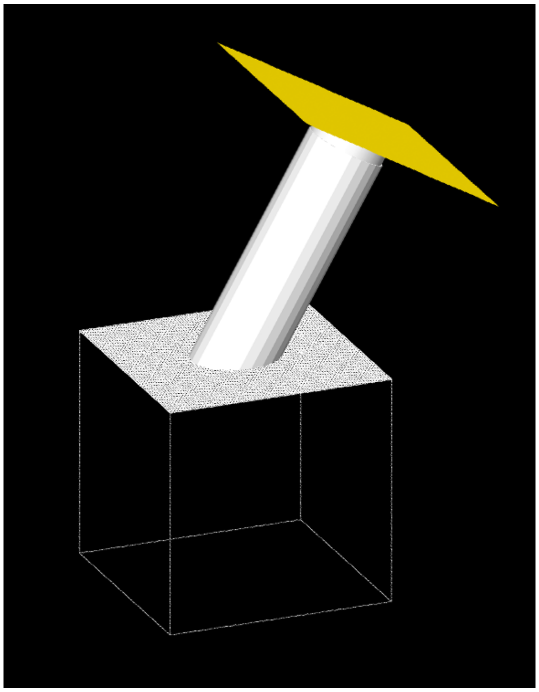

Figure S1. Simulation representation for non-reference 30°-beveled applicator, 10 cm in diameter. The phase-space is positioned on top of the applicator and the voxelized phantom outline is show underneath the applicator.

Applicator diameter [cm]	Bevel	OF_{field}	Energy 6 MeV OF	6 MeV RD	9 MeV OF	9 MeV RD	12 MeV OF	12 MeV RD
8	0°	Commissioning	1.032		1.051		1.062	
		Mills et al.	1.043	1.1%	1.050	0.2%	1.070	0.8%
		Simulation	1.053 ± 0.012	2.1%	1.075 ± 0.019	2.3%	1.072 ± 0.030	0.9%
	15°	Commissioning	1.007		1.034		1.062	
		Mills et al.	-	-	-	-	-	-
		Simulation	-	-	1.035 ± 0.040	0.1%	-	-
	30°	Commissioning	0.981		1.004		1.029	
		Mills et al.	-	-	-	-	-	-
		Simulation	0.964 ± 0.052	1.7%	-	-	1.004 ± 0.021	2.4%

Table S1. Field output factor (OF_{field}) comparison. A total of 36 OFs, corresponding to the most common applicator configurations and small field sizes, were estimated using the Monte Carlo (MC) simulation. Only a subset of the OFs measured during commissioning is listed here. The deviation from the commissioning output factor is expressed as $RD = 100 \cdot \left|1 - OF/OF_{Commissioning}\right|$ (the relative deviation in %) for both the OFs obtained by Mills et al.[1] and those from the simulation. The table continues on the next page.

Applicator diameter [cm]	Bevel	OF_{field}	Energy 6 MeV OF	6 MeV RD	9 MeV OF	9 MeV RD	12 MeV OF	12 MeV RD
7	0°	Commissioning	1.033		1.066		1.085	
		Mills et al.	1.005	2.7%	1.050	1.5%	1.070	1.4%
		Simulation	1.065 ± 0.039	3.1%	1.060 ± 0.025	0.6%	1.106 ± 0.027	1.9%
	15°	Commissioning	1.013		1.048		1.087	
		Mills et al.	-	-	-	-	-	-
		Simulation	1.050 ± 0.041	3.6%	-	-	-	-
	30°	Commissioning	0.98		1.018		1.053	
		Mills et al.	-	-	-	-	-	-
		Simulation	0.995 ± 0.042	1.5%	1.033 ± 0.036	1.5%	1.046 ± 0.038	0.7%
6	0°	Commissioning	1.100		1.147		1.171	
		Mills et al.	1.085	1.4%	1.159	1.0%	1.172	0.1%
		Simulation	1.060 ± 0.053	3.6%	1.114 ± 0.039	2.9%	1.121 ± 0.035	4.3%
	15°	Commissioning	1.074		1.127		1.173	
		Mills et al.	-	-	-	-	-	-
		Simulation	-	-	1.093 ± 0.034	3.0%	-	-
	30°	Commissioning	1.037		1.093		1.133	
		Mills et al.	1.027	1.0%	1.105	1.1%	1.113	1.8%
		Simulation	1.008 ± 0.027	2.8%	1.073 ± 0.019	1.8%	1.078 ± 0.030	4.8%
5	0°	Commissioning	1.076		1.14		1.177	
		Mills et al.	1.064	1.1%	1.162	1.9%	1.195	1.5%
		Simulation	1.027 ± 0.038	1.0%	1.134 ± 0.026	0.5%	1.148 ± 0.030	2.4%
	15°	Commissioning	1.054		1.126		1.181	
		Mills et al.	0.961	8.8%	-	-	-	-
		Simulation	1.035 ± 0.024	3.8%	-	-	-	-
	30°	Commissioning	1.021		1.095		1.145	
		Mills et al.	0.961	5.9%	1.063	2.9%	1.150	0.4%
		Simulation	0.995 ± 0.028	2.5%	1.058 ± 0.032	3.4%	1.067 ± 0.042	6.8%
4	0°	Commissioning	1.013		1.104		1.153	
		Mills et al.	1.001	1.2%	1.123	1.7%	1.202	8.8%
		Simulation	1.002 ± 0.043	1.1%	1.089 ± 0.033	1.4%	1.158 ± 0.027	4.9%
	15°	Commissioning	0.997		1.089		1.16	
		Mills et al.	-	-	-	-	-	-
		Simulation	-	-	-	-	-	-
	30°	Commissioning	0.971		1.063		1.116	
		Mills et al.	0.889	8.4%	1.009	5.1%	1.110	0.5%
		Simulation	0.942 ± 0.028	3.0%	1.067 ± 0.049	0.3%	1.115 ± 0.035	0.1%
3.5	0°	Commissioning	0.965		1.076		1.129	
		Mills et al.	-	-	-	-	-	-
		Simulation	0.941 ± 0.019	2.5%	1.063 ± 0.020	1.2%	1.135 ± 0.029	0.5%
	15°	Commissioning	0.940		1.053		1.151	
		Mills et al.	-	-	-	-	-	-
		Simulation	-	-	-	-	-	-
	30°	Commissioning	0.917		1.03		1.097	
		Mills et al.	-	-	-	-	-	-
		Simulation	-	-	-	-	-	-

Table S1. Continuation.

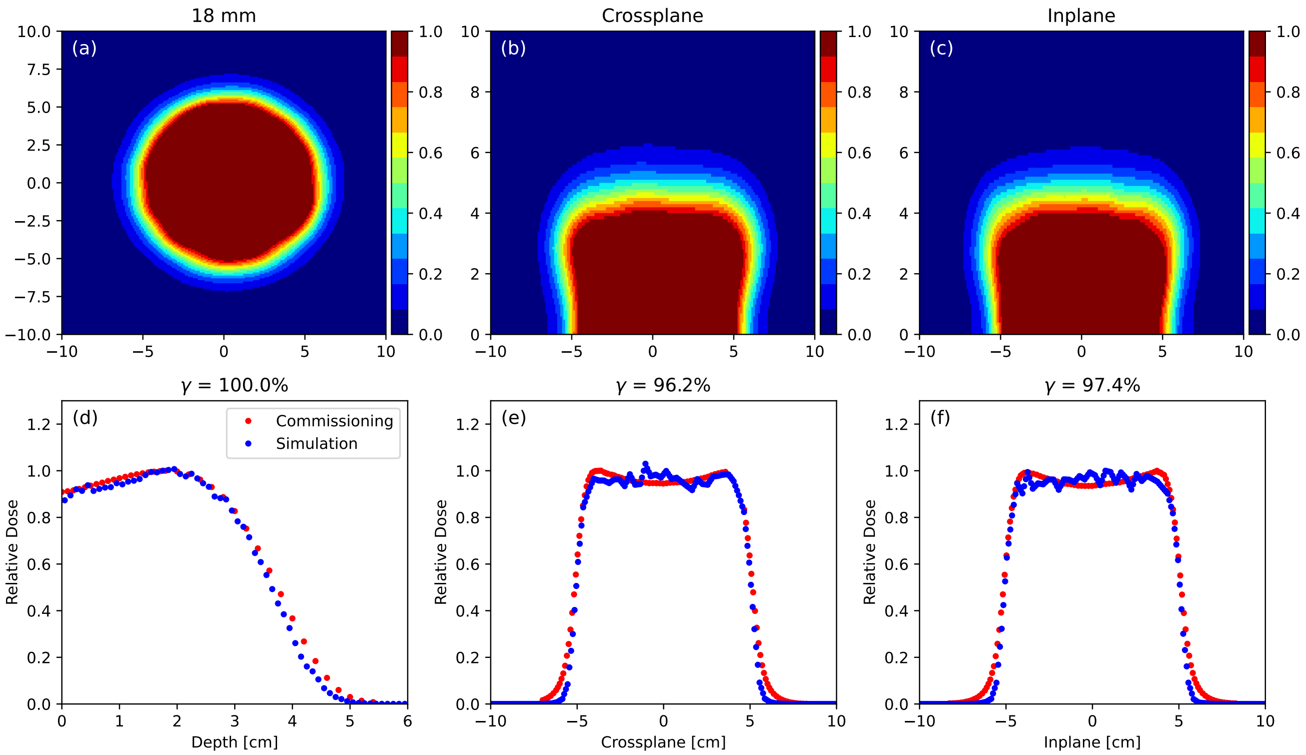

Figure S2. Simulation output for a 9 MeV electron beam. Simulated water phantom dose distribution for the reference 10 cm 0°-bevel applicator (blue) and experimentally measured data (red). The isodose distributions are normalized to the central axis dose at $z_{max} = 18\,\mathrm{mm}$ [center in **(a)**]. Isodose distributions in the inplane and crossplane planes are shown in **(b)** and **(c)**, along with their dose profiles at z_{max} in **(e)** and **(f)**, respectively. The PDD is shown in **(d)**. The global gamma pass rate for the 3 mm/3% criterion is shown above the curves. The displayed data accounts for 25×10^6 simulated primary electrons.

References

1. Mills, M. D., Fajardo, L. C., Wilson, D. L., Daves, J. L. & Spanos, W. J. Commissioning of a mobile electron accelerator for intraoperative radiotherapy. *J. Appl. Clin. Med. Phys.* **2**, 121–130, DOI: 10.1120/jacmp.v2i3.2605 (2001).
